# Data on the use of optional ergative case marking in Tujia, an endangered language of southwest China

**DOI:** 10.1016/j.dib.2019.104019

**Published:** 2019-06-26

**Authors:** Man Lu, Jeroen van de Weijer, Yu Ma, Zhengguang Liu

**Affiliations:** a School of Foreign Languages, Shenzhen University, Nanhai Ave. 3688, Shenzhen, Guangdong Province, China; b Foreign Language School, Hunan University, Lushan South Road, No. 2, Hunan Province, Changsha City, China

## Abstract

Agents are optionally marked in Tujia, an endangered language spoken in southwest China. Sentences, discourse texts and conversations are presented in this dataset.

**Table:** Specifications table

Subject area	*Linguistics*
More specific subject area	*Use of the optional ergative marker ko*^*35*^*in Tujia*
Type of data	*Tables, sentences, conversations*
How data was acquired	*Field work; audio recorded on Sony ICD-SX1000; video recorded on Sony HDR-CX680*
Data format	*Glossed with translations (raw)*
Experimental factors	*Random sample of sentences, texts, or self-narratives.*
Experimental features	*Illustration, translation, self-narrative*
Data source location	*Dianfang town, Baoge village, Longshan county* *Changsha, China*
Data accessibility	*All the data are included in this data article.*
Related research article	Man Lu, Jeroen van de Weijer, Chris Sinha, Zhengguang Liu. 2019. Optional Ergative Marking in Tujia (*Lingua* (223), 46–66).

**Table:** 

**Value of the data** • The dataset can serve as a reference for the use of the optional ergative marker in Tujia. • The dataset can be used to investigate the semantics, morphology and grammar of the endangered Tujia language. • The dataset can provide insight in the customs and culture of Tujia. • The reference section can serve as a useful resource for researchers in these areas.

## 1 Data

The data contained in this article are listed as below. The dataset consists of sentences with or without the ergative case marker. Sentences are classified according to degree of clausal transitivity and aspect. Other constructions illustrated include the imperative construction and the resultative construction. Secondly, excerpts from conversations are included in this data article. Finally, this data article contains data from some daily conversations by local natives (see Table 1, Table 2, Table 3). Tables showing the statistical analysis of these datasets are also included. For interpretation and analysis of the data, see Man et al. [1].

**Table 1:** Frequency of use and non-use of the ergative in Tujia narratives.

Clause type	Use of ergative	Percentages of use of ergative
Intransitive nominals	13	12%
Transitive nominals	142	37%
Ditransitive nominals	151	40%
Total	306

**Table 2:** Frequencies of use and non-use of ergative across different age groups.

Age	Use of ko^35^	Non-use of *ko*^35^
over 70	1005(56%)	795
60–69	825(46%)	975
younger than 60	495(28%)	1305

**Table 3:** Frequency of use and non-use of the ergative in perfective.

Aspect	Use of ergative	Non-use of ergative
Perfective	61(61%)	39
Imperfective	9(9%)	91

## 2 Experimental design, materials and methods

The data were collected in four fieldwork trips during 2014–2018. The superscript values indicate tone contours.

### 2.1 Non-canonical word order^1^

**Table:** 

(1)	zo^35^	li^35^	*(**ko**^**35**^）	ka^35^	liau^21^.
	goat	tiger	KO	eat	PERF
	‘The goat was eaten by a tiger.’

**Table:** 

(2)	la^21^ tse^55^tsi^21^	*(**ko**^**35**^)	ia^53^	si^21^	liau^21^.
	road car	KO	squeeze	break	PERF
	‘The road has been squeezed broken by cars.’

**Table:** 

(3)	na^53^mi^21^	ze^35^su^53^ *(**ko**^**35**^)	ts^h^ui^55^ ia^53^	liau^21^	tong^35^	liau^21^.	
	door	wind	KO	blow	PERF	open	PERF
	‘The door was blown open.’

**Table:** 

(4)	ko^35^ mie^35^t^h^a^55^ ts^h^e^21^	*(**ko**^**35**^)	sa^35^	liau^21^.
	3SG thunder	KO	strike	PERF
	‘He was stricken by thunder.’

**Table:** 

(5)	an^53^ŋai^53^no^35^ti^53^	*(**ko**^**35**^)	xa^21^	liau^21^.
	younger brother	KO	beat	PERF
	‘(My) younger brother was beaten by someone.’			

### 2.2 Transitive

#### 2.2.1 Verbs in low transitivity

**Table:** 

(6)	p^h^a^55^p^h^u^21^	(**ko**^**35**^)	na^53^mi^21^	sa^21^	liau^21^.
	grandfather	KO	door	lock	PERF
	‘Grandfather locked the door.’

**Table:** 

(7)	ŋa^35^	p^h^u^21^ɲi^21^	pai^21^	xa^21^	liau^21^.
	1SG	yesterday	card	play	PERF
	‘I played cards yesterday.’

**Table:** 

(8)	ŋa^35^	p^h^u^21^ɲi^21^	tian^21^in^21^	pa^21^ liau^21^.
	1SG	yesterday	movie	see PERF
	‘I saw a movie yesterday.’

**Table:** 

(9)	ko^35^	ian^55^	nau^53^	p^h^u^35^	liau^21^.
	3SG	cigarette	one.CL	buy	PERF
	‘She bought a pack of cigarette.’

**Table:** 

(10)	ko^35^	ian^55^	xo^35^lan^21^	p^h^u^35^	liau^21^.
	3SG	cigarette	many	buy	PERF
	‘She bought many packs of cigarette.’

**Table:** 

(11)	ko^35^	p^h^u^21^ɲi^55^	t^h^i^53^ k^h^i^21^	liau^21^	tɕi^21^	la^53^k^h^o^21^	liau^21^.
	3SG	yesterday	tumble	PERF	leg	break	PERF
	‘She tumbled and broke her leg.’

**Table:** 

(12)	ŋa^35^	nai^35^	li^35^	nau^53^	i^21^.
	1SG	today	tiger	one.CL	see
	‘I saw a tiger today.’

**Table:** 

(13)	ŋa^35^	**ko**^35^	kan^21^	ku^53^	i^21^	liau^21^.
	1SG	3SG	mountain on		see	PERF
	‘I saw him on the mountain.’

**Table:** 

(14)	ŋa^35^	(**ko**^35^)	lo^35^	na^55^ xu^33^	i^21^	po^21^ la^21^.
	1SG	KO	person	one CL	see	EXP
	‘I once saw this person.’

**Table:** 

(15)	a.	ta^21^ɲie^55^	(**ko**^35^)	ɕi^55^pa^55^	za^21^	liau^21^.
		mother	KO	clothes	wash	PERF
		‘My mother washed clothes.’

**Table:** 

b.	a^21^k^h^o^55^	tian^35^ in^53^	pa^21^	liau^21^.
	older brother	movie	see	PERF
	‘My older brother saw a movie.'

**Table:** 

(16)	ŋa^35^	lai^55^	san^21^	k^h^o^53^	liau^21^.
	1SG	today	teach	class	PERF
	‘I taught classes today.’

**Table:** 

(17)	tian^21^ tɕi^53^	ts^h^i^55^p^h^u^55^	pa^53^	liau^21^.
	person name	book	read	PERF
	‘Tian ji read (a) book.’

**Table:** 

(18)	tian^21^ tɕi^53^	ai^55^	ts^h^i^55^p^h^u^55^	na^55^	pu^21^	pa^53^ liau^21^.
	person name	DEM	book	one	CL	read PERF
	‘Tian tɕi read that book.’

**Table:** 

(19)	tian^21^ tɕi^53^	(ko^35^)	ai^55^	ts^h^i^55^p^h^u^55^	na^55^pu^21^	pa^53^	tɕi^21^	liau^21^.
	person name	KO	DEM	book	one.CL	read	finish	PERF
	‘Tian tɕi finished reading that book.’

**Table:** 

(20)	a.	a^55^ŋai^55^	zi^21^	liau^21^.
		younger brother	cry	PERF
		‘My younger brother cried.’

**Table:** 

b.	a^55^ŋai^55^	（**ko**^**35**^）	xa^21^wa^55^	zi^21^	liau^21^.
	younger brother	KO	loudly	cry	PERF
	‘Younger brother cried loudly.’

**Table:** 

(21)	a^55^ŋai^55^	（**ko**^**35**^）	na^53^mi^21^	xa^21^	liau^21^.
	younger	KO	door	knock	PERF
	‘(My) younger brother knocked on the door.’

**Table:** 

(22)	a.	a^21^se^55^	(ko^35^)	na^53^mi^21^	xa^21^	la^55^?
		who	KO	door	knock	PROG
		‘Who is knocking on the door lightly?’

**Table:** 

b.	a^55^ŋai^55^	（**ko**^**35**^）	ka^35^ki^53^li^53^li^53^	mo^21^	na^53^mi^21^	xa^21^	la^55^.
	younger	KO	lightly	ADV	door	knock	PROG
	‘(My) younger brother is knocking on the door.’

**Table:** 

(23)	kai^35^	ts^h^i^55^p^h^u^55^	āŋa^35^	（**ko**^**35**^）	ɲie^35^ tɕie^53^	pa^21^	liau^21^.
	DEM	book	1SG	KO	two time	read	PERF
	‘I read this book twice.’

**Table:** 

(24)	ɕian^35^	p^h^o ^35^ka^55^	(**ko**^**35**^）	an^35^ ɲie^55^	pan^55^tsu^55^zen^21^	t^h^au^35^.
	family name	teacher	KO	1PL	tutor	NEG.PERF
	‘Teacher Qian is not our tutor any longer.’

**Table:** 

(25)	a.	ŋa^35^	ɲie^35^	ɕi^21^pa^53^	za^21^	liau^21^	ma^21^?
		My	GEN	clothes	wash	PERF	QM
	‘Have my clothes been washed?’

**Table:** 

b.	a^35^ta^55^	（**ko**^**35**^）	ɕi^21^pa^53^	za^21^	liau^21^.
	sister	KO	clothes	wash	PERF
	‘(My) sister had washed the clothes.’

**Table:** 

(26)	ŋa^35^	le^55^	a^55^se^21^	(ko^35^)	ɲi^35^	kan^55^	ɣa^21^！
	1SG	except	who	KO	2SG dare		marry
	‘Who except me dare to marry you?’

**Table:** 

(27)	A.	ɲi^35^	nai^35^ xou^35^	la^55^tɕie^53^ɕi^21^	ka^35^	liau^21^?^2^
		2SG	this morning	what	eat	PERF
		‘What did you eat this morning?’

**Table:** 

B.	ŋa^35^	pao^33^ku^33^	ka^35^	liau^21^.
	1SG	corn	eat	PERF
	‘I ate corn.’

**Table:** 

(28)	A.	ki^21^tse^53^	ɲi^21^	ɲie^53^	a^21^ko^35^?
		where	2SG	GEN	brother
		‘Where is your brother?’

**Table:** 

B.	ɲa^35^ a^21^ ɲie^53^	（**ko**^**35**^）	ts^h^e^35^	xa^21^ lu^21^.
	my mother	KO	vegetable	pick PERF.DIR
	‘My mother has gone to pick vegetables.’

**Table:** 

(29)	A.	ɲi^35^	（**ko**^**35**^） kai^35^	no^35^	xau^55^ɕi^55^	xo^21^	t^h^ai^21^?
		2SG	KO	DEM	person	know	or NEG
		‘Do you know this person or not’?

**Table:** 

B.	ŋa^35^	ko^35^	zen^35^tai^55^	ɕi^21^.
	1SG	3SG	know	NMLZ
	‘I know him.’

**Table:** 

(30)	A.	ko^35^	an^35^ɲie^55^	ɕiau^35^ t^h^aŋ^21^	ɲie^21^	en^21^tsi^21^	ɲie^21^ ɕian^53^	p^h^o^35^ ka^21^.
		3SG	1PL	school	GEN	come	REL family name	teacher
		‘He is teacher Xiang who has just come to our school.’

**Table:** 

B.	ko^35^	(**ko**^**35**^**)**	ɕiau^35^t^h^an^21^	je^55^	lie^21^	ka^55^	ɲie^55^	liau^21^.
	3SG	KO	school	arrived	PERF	Several	days	PERF
	‘He arrived at our school several days ago.’

**Table:** 

(31) A.	ɕian^35^	p^h^o^35^ka^55^	(**ko**^**35**^)	an^35^ȵie^55^ ɕiu^35^ tɕiau^21^	tɕiau^55^	la^55^	ma^35^?
	Family name	teacher	KO	1PL math	teach	PROG	QM
	‘Is teacher Xiang teaching us math?’

**Table:** 

B.	ɕian^35^ p^h^o^35^	ka^55^	(**ko**^**35**^)	an^35^ ȵie^55^	ɕiu^35^ ɕiau^21^	tɕiau^55^	la^55^.
	family name	teacher	KO	1PL	math	teach	PROG
	‘Teacher Xiang teaches us math.’

**Table:** 

(32)	Wang^35^	p^h^o^35^ ka^55^	(**ko**^**35**^)	an^35^ ȵie^55^	ɕiu^35^ɕiau^21^	tɕiau^55^	liau^21^.
	family name	teacher	KO	1PL	math	teach	PERF
	‘Teacher Xiang will not teach us math any longer.’

**Table:** 

(33)	ȵi^35^	（**ko**^**35**^)	ŋa^35^	a^21^pa^53^	ɣɨ^21^	liau^21^	ma^35^?
	2SG	KO	1SG	father	see	PERF	QM
	‘Have you seen my father?’						

#### 2.2.2 Verbs in high transitivity

**Table:** 

(34)	A.	a^21^pa^55^	（ko^35^）	a^21^ko^21^	xa^21^	liau^21^?
		father	KO	older brother	beat	PERF
		‘Did father beat older brother?’

**Table:** 

B.	ko^35^	xa^21^	ta^35^.
	3SG	beat	NEG
	‘He did not beat (him).’

**Table:** 

(35)	ko^35^	(**ko**^**35**^)	ŋa^35^	ts^h^a^53^	liau^21^.
	3SG	KO	1SG	cheat	PERF
	‘He cheated me.’

**Table:** 

(36)	a^21^pa^55^	（**ko**^**35**^）	ka^21^ tsi^21^	xo^21^lie^21^	u^53^	xa^21^	se^21^	liau^21^.
	father	KO	stick	INSTR	snake	beat	die	PERF
	‘My father killed the snake with a stick.’

**Table:** 

(37)	p^h^o^35^ ka^55^	(**ko**^**35**^)	ŋa^35^	lo^21^	liau^21^.
	teacher	KO	1SG	criticize	PERF
	‘The teacher criticized me.’

**Table:** 

(38)	a^21^pa^55^	(**ko**^**35**^)	ka^21^	u^53^	lu^21^.
	father	KO	wood	cut	DIR
	‘My father has gone to cut wood.’

**Table:** 

(39)	ŋa^35^	xi^53^	po^55^ la^55^	ɲi^35^	li^35^	lau^53^	xa^21^	se^21^	liau^21^.
	1SG	hear	PERF	2SG	tiger one.CL		beat	die	PERF
	‘I heard that you beat a tiger to die.’

**Table:** 

(40)	kei^55^ tse^55^	(**ko**^**35**^**)**	lo^53^tie^35^k^h^a^55^	la^21^xa^21^mo^21^suŋ^55^	k^h^o^55^tɕi^35^	lu^21^.
	3PL	KO	enemy	bad	chase	DIR
	‘They had expelled the enemy.’					

## 3 Dative construction

**Table:** 

(41)	p^h^a^55^ p^h^u^21^	（ko^35^）	ŋa^35^	po^53^	sa^21^lie^35^pu^21^	li^55^	liau^21^.
	grandfather	KO	1SG	DAT	story	tell	PERF
	‘My grandfather told me a story.’

**Table:** 

(42)	a^21^ta^55^	（ko^35^）	ŋa^35^	po^53^	ɕi^55^pa^55^	lie^35^p^h^i^55^	p^h^u^55^liau^21^.
	old sister	KO	1SG	DAT	clothes one CL		buy PERF
	‘(My) older sister bought a piece of clothes for me.’

**Table:** 

(43)	ŋa^35^	xa^55^ts^h^e^55^	po^55^	se^21^	t^h^u^35^.
	1SG	vegetable	DAT	fertilizer	put
	‘I put the fertilizer on the vegetables.’

**Table:** 

(44)	ŋa^35^	(**ko**^**35**^）	a^55^ko^55^	ts^h^i^55^p^h^u^55^	la^35^ p^h^u^53^	lie^21^	liau^21^.
	1SG	KO	brother	book	one.CL	send	PERF
	‘I sent a book to my brother.’

**Table:** 

(45)	a^21^ta^55^	（**ko**^**35**^）	ŋa^35^	po^35^	ku^55^s^h^i^21^	nau^53^	ze^21^	liau^21^.
	older sister	KO	1SG	DAT	flower	one.CL	give	PERF
	‘My older sister gave me a flower.’

**Table:** 

(46)	uang^35^	p^h^o^35^ka^21^	（**ko**^**35**^）	a^35^ ta^53^	tshu^35^	po^21^	shi^21^ liau^21^ .
	family name	teacher	ERG	old sister	home	GOAL	sent PERF
	‘Teacher Wang sent my old sister home.’						

## 4 Inanimate argument

**Table:** 

(47)	ts^h^ao^21^io^53^	* (ko^35^)	ko^35^	ȵie^53^	pin^53^	tsa^21^	liau^21^.
	herb	KO	3SG	GEN	disease	cure	PERF
	‘Herbs cured his disease.’

**Table:** 

(48)	xu^21^p^h^a^21^ts^h^ie^21^	*(ko^35^)	a^55^ȵie^53^	ts^h^e^21^ka^53^	ian^55^	liau^21^.
	river water	KO	1PL	field	drown	PERF
	‘River water had drowned our field.’					

## 5 Intransitive

**Table:** 

(49)	pu^55^ts^h^o^21^	tsau^21^ .
	guest	leave
	‘The guest has left.’

**Table:** 

(50)	kei^55^tse^55^	ɲie^53^	liau^21^ .
	3PL	laugh	PERF
	‘They laughed.’

**Table:** 

(51)	kai^35^ xa^21^lie^21^	**ko**^35^	se^35^ liau^21^ .
	DEM dog	*KO /3SG	die PERG
	‘That dog died.’

**Table:** 

(52)	a^21^ɲie^55^	loŋ^55^	a^55^lie^55^	na^35^	loŋ^55^ liau^21^.
	mother	cough	after	one	year PERF
	‘My mother has been coughing for one year.’

**Table:** 

(53)	a.	p^h^a^55^ p^h^u^21^	s e^35^	liau^21^.
		grandfather	die	PERF
		‘(My grandfather) died.’

**Table:** 

b.	p^h^a^55^ p^h^u^21^_,_	ko^35^	s e^35^ liau^21^ .
	grandfather	*KO/3SG	die PERF
	‘(My grandfather) died.’		

## 6 Telic events

### 6.1 Telic events

**Table:** 

(54)	ɲi^35^	（ko^35^）	ŋa^35^	ɲie^21^	a^21^pa^53^	pa^35^	liau^21^	ma?
	2SG	KO	1SG	GEN father		see	PERF	SFP
	‘Did you see my father?’							

### 6.2 Resultative construction

**Table:** 

(55)	ŋa^35^	lo^21^ ka^21^ɲi^35^	(ko^35^)	si^35^thiǝ^35^	ma^35^	liau^21^	so^35^so^35^li^21^li^21^.
	my	wife	KO	desk	clear	PERF	clean clean
	‘My wife cleaned the desk very clean.’

**Table:** 

(56)	loŋ^21^kui^35^	(**ko**^**35**^)	zo^35^	tsia^55^	lie^55^	la^21^	lu^21^.
	person name	KO	goat chase		PERF	walk	PERF.DIR
	‘Longgui chased the goat away.’

**Table:** 

(57)	pã^35^	(**ko**^**35**^)	sa^55^	lau^55^	piǝ^55^	lu^21^
	eagle	KO	duck	one.CL	seize	PERF.DIR
	‘One eagle seized a duck and took it away.’

**Table:** 

(58)	sa^55^ wo^21^ nũ^21^, ŋa^35^	**(ko**^**35**^) sa^33^	nũ^55^ xa^21^ se^21^ .	liau^21^
	duck five CL 1SG	KO three	CL beat die	PERF
	‘Five ducks, I beat three of them to death.’

**Table:** 

(59)	ɲi^35^	(**ko**^**35**^)	u^53^	xa^21^	se^21^ liau^21^.
	2SG	KO	snake	beat	die PERF
	‘You have beat the snake to death.’

**Table:** 

(60)	ŋa^35^ (**ko**^**35**^)	p^h^in^53^ko^21^ no^35^tei^53^ tso^21^	p^h^u^21^	liau^21^.
	1SG KO	apple bag	put	PERF
	‘I put the apples in the bag.’

**Table:** 

(61)	a^55^ma^55^	ɣe^35^	liǝ^53^	lou^21^xo^21^	liau^21^
	grandma	walk	LNK	tired	PERF
	‘My grandma became tired from walking.’

**Table:** 

(62)	a^55^ta^55^ (**ko**^**35**^)	si^35^t^h^iǝ^35^	ma^35^	liau^21^	so^35^so^35^li^21^li^21^.
	sister KO	desk	clean	PERF	clean clean
	‘My sister had cleaned the desk very clean.’

**Table:** 

(63)	sɨə^35^	(ko^35^)	si^55^ɲi^21^t^h^u^21^ka^21^	wu^35^ts^h^o^53^	o^35^t^h^u^55^	si^35^lie^55^pie^21^!
	2PL	KO	matchmaker	cow stable	into	shut
	‘You shut the match-maker into the cow stable!’ (Yao 2013: 129)

**Table:** 

(64)	ɲi^35^	(**ko**^**35**^)	kai^35^ io^35^	ze^35^	le^21^!
	2SG	KO	his pill	swallow	SFP
	‘You swallow this pill!’

**Table:** 

(65)	ɲi^35^	ɲia^53^	xu^21^	koŋ^53^se^35^	xuei^35^	k^h^ai^53^
	2SG	two	CL	commune hall	conference	attend
	‘You two go to the commune hall to attend the conference.’

**Table:** 

(66)	ko^35^	(**ko**^**35**^)	tɕ^h^in^53^mo^21^	no^53^	tsu^55^	oŋ^21^	tie^35^	kuã^35^	o^55^	a^21^ɲie^53^?
	3SG	KO	how	people	home live		used	to	EXC	mother
	‘How can she get used to living in (other) people's home, mother?’

**Table:** 

ŋa^35^	(**ko**^**35**^)	ɕ^h^in^53^mo^21^ no^53^	tsu^55^	ũ-i^35^	o^55^	a^21^ ɲie^53^?
	1SG KO	how	people home	stay-FUT	EXC	mother
	‘How could I stay in a stranger's home, mother?’

**Table:** 

ŋa^35^	(ko^35^)	ɕ^h^in^53^mo^21^	ɲie^53^	k^h^a-i^35^	o^55^	a^21^ɲie^53^?
1SG	KO	how	live	life-FUT	EXM	mother
	‘How could I live my life, mother? ’ (From a song of *Crying Marriage*)					

## Footnotes

1 Abbreviations and glosses: 1–3.SG-PL = Person.Number; CL = Classifier; DAT = Dative; DEM = Demonstrative; DIR = Direction; EXM = Exclamative marker; EXP = Experiencer; GEN = Genitive; GOL = Goal; LNK = linker; NEG = Negation; NMLZ = Nominalizer; PERF = Perfective; PL = Plural; PROG = Progressive; QM = Question marker.

2 Capital A and B are used to indicate participants in a conversation.
