# Supplementary material for: Data on the use of optional ergative case marking in Tujia, an endangered language of southwest China
Source: Data Brief. 2019 Jun 26;26:104019. doi: 10.1016/j.dib.2019.104019 (PMC6811877; doi:10.1016/j.dib.2019.104019)
Supplement: Multimedia component 1 [file mmc1.docx]

There are no conflict of interests.
